# Accuracy of owner-reported diagnoses for dogs enrolled in the Dog Aging Project as compared to veterinary electronic medical records

**DOI:** 10.1371/journal.pone.0342427

**Published:** 2026-03-04

**Authors:** Sarah M. Schmid, Courtney L. Sexton, Alexandria Yoerger, Mandy Kauffman, Robyn L. McClelland, D.A.P. Consortium, Kate E. Creevy, Audrey Ruple

**Affiliations:** 1 Department of Small Animal Clinical Sciences, College of Veterinary Medicine, University of Tennessee, Knoxville, Tennessee, United States of America; 2 Department of Population Health Sciences, Virginia-Maryland College of Veterinary Medicine, Blacksburg, Virginia, United States of America; 3 Department of Small Animal Clinical Sciences, College of Veterinary Medicine & Biomedical Sciences, Texas A&M University, College Station, Texas, United States of America; 4 Center for Studies in Demography and Ecology, University of Washington, Seattle, Washington, United States of America; 5 Department of Biostatistics, University of Washington, Seattle, Washington, United States of America; National Veterinary Research Institute (NVRI), NIGERIA

## Abstract

The objective of this project was to evaluate the accuracy of owner-reported health diagnoses in dogs compared to veterinary electronic medical records (VEMRs) using data from the Dog Aging Project (DAP), a longitudinal study of companion dogs in the United States. We hypothesized that owner-reported diagnoses would align more closely with VEMRs for acute or emergent conditions and less so for benign or self-limiting conditions. A subsample of 350 dogs was selected from the 2020 curated DAP dataset (n = 27,541). Dogs were included if they had VEMRs covering ≥ 85% of their life up to the time of survey completion. Forty-one dogs were excluded due to incomplete VEMRs, resulting in a final sample of 309 dogs included in this observational study comparing owner responses in the Health and Life Experience Survey (HLES) (https://github.com/dogagingproject/dataRelease/tree/master/SurveyInstruments/HLES) to VEMR data abstracted by masked reviewers. VEMR reviewers completed a survey mirroring HLES and identified supporting evidence for each diagnosis. Agreement between owner-reported and VEMR-verified diagnoses was assessed across 20 disease categories, with a focus on five target disease categories (TDCs). Agreement between owner and VEMR data was ≥ 90% in 10 of 20 disease categories. Agreement was highest in endocrine, immune, and “other” categories (99%) and lowest in dental/oral (44%). Among TDCs, orthopedic and traumatic conditions had higher agreement (>78%), while dermatologic and dental/oral categories showed the least. Owner-reported dog health data showed substantial concordance with VEMRs for many disease categories, supporting their utility in research. Thus, owner-reported diagnoses might provide a reliable and scalable supplement or alternative to VEMRs in veterinary research and epidemiology.

## Introduction

Conducting veterinary research that endeavors to improve real-world health outcomes for animals depends on access to large quantities of diverse and, ideally, longitudinal data. Veterinary electronic medical records (VEMRs) are generally considered the gold standard source for collecting a crucial segment of these data in companion animals, specifically clinical phenotypes [1–6]. While there are indeed many advantages to using VEMRs to extract health-related phenotypes, there remain persistent challenges when trying to extract consistent and compatible phenotype data for many patients due to differences in interface operability and standardization [7]. Differing identification requirements across systems and lower accessibility to systems (e.g., due to cost) compared to human EMRs [8], along with the lack of a unifying system for standardizing diagnoses across providers and institutions, such the International Classification of Diseases (ICD) codes used in human medicine [9], present additional complications when using VEMRs as a source for data extraction.

Furthermore, compared to hospital-based veterinary practices and those involved in medical professional training, independent veterinary practices are less likely to utilize a VEMR system [10], therefore potentially omitting a substantial source of data from large studies. Still, despite these current obstacles, VEMRs demonstrate the potential for expanding on veterinary informatics and are among the most comprehensive sources of medical information for pets, especially when it comes to data that can advance the efficacy of the research-to-real world practice pipeline.

When compared to VEMRs, the potential benefits of using multi-source surveys to collect pet health-related data include the ease and speed of data acquisition, data standardization, and customization potential. Survey data have been successfully utilized in numerous studies aiming to find associations between phenotypes of interest (e.g., physical traits, lifestyle factors) and other paired data (e.g., genetic data, health outcomes). For example, similar to human health survey data being successfully used to identify genetic associations for hair color, eye color, and freckling in humans [11], Embark Veterinary, Inc. used owner-reported phenotype data combined with canine genotype data from high-density single nucleotide polymorphism arrays to detect a locus associated with blue eye color in dogs [12].

However, survey data can also present their own challenges and limitations. For example, these data can be prone to error, including measurement variability, as when respondent answers do not correspond directly with the intended question from the researcher due to factors such as poor survey design, decreasing motivation of respondents during long surveys, or inability of a respondent to remember the correct answer [13]. In humans, accuracy of self-reported data compared to EMR data has also been shown to vary with the perceived social desirability of one answer over another [13,14]. For example, studies assessing human cancer screening procedures indicate that self-reporting tends to overestimate the population that has been screened [1,5,6]. Despite these limitations, self-administered health surveys have shown satisfactory concordance with EMR data in several settings and populations including self-reports of injuries and various chronic conditions [2,4,15,16].

Such concordance among human data sources could be encouraging from a veterinary perspective if the same holds true for VEMRs and owner-reported information about their pets, as there is a high dependency on such reports among clinicians and researchers alike. Furthermore, integrating available VEMR data with novel owner-reported survey responses can enhance both the quantity and diversity of information available to support a wide range of clinical and research needs across veterinary fields.

Recognizing that access to both types of data is essential for advancing our understanding of disease in dogs, the Dog Aging Project (DAP), a longitudinal study of aging and age-related disease in US companion dogs, gathers VEMR data alongside survey data [17]. Consequently, the DAP provides a unique opportunity to compare owner-reported data to VEMR data for the same individuals and health-related phenotypes. To assess the accuracy and validity of owner-reported pet health data, the aim of this study was to compare the concordance of owner-reported health diagnoses with VEMR data among a cohort of dogs enrolled in the DAP. We hypothesized that dog-owners would be in highest concordance with VEMRs when reporting diagnoses that were acute or emergent (e.g., traumatic bone fracture) or chronic (e.g., hypothyroidism requiring daily medication) and less concordant when describing more innocuous diagnoses (e.g., flank alopecia, grade 1 dental disease, or self-limiting vomiting).

## Methods

### Data collection

The DAP is a community science project in which owners enroll their companion dogs through a series of online surveys [17]. Owners who opt to enroll their dogs in the study are first led through an extensive written informed consent process. Once consent is confirmed, all owners complete a Health and Life Experience Survey (HLES) which collects information on the dogs’ signalment, behavior, environment, physical activity, diet, and health. As part of the HLES, each participant is asked to identify their primary care veterinarian and indicate whether they are willing to share their dogs’ medical records with the DAP for research purposes. Participants who express willingness to share their dogs’ medical records are invited to obtain VEMRs from their veterinarian and submit them through electronic file transfer. Once uploaded, the VEMR is evaluated by DAP personnel to ensure it meets the standard for eligibility and to capture the dates for which the VEMR entries are recorded [18]. The study data from DAP are collected and managed using REDCap (Research Electronic Data Capture) tools hosted at the University of Washington [19,20].

As an open data project, DAP survey instruments are made publicly available on GitHub: https://github.com/dogagingproject/dataRelease/tree/master/SurveyInstruments. The University of Washington Institutional Review Board (IRB) deemed that recruitment of dog owners for the DAP, and the administration and content of each AP questionnaire, are human subjects research that qualifies for category 2 exempt status (IRB ID no. 5988, effective 10/30/2018). No interactions between researchers and privately owned dogs occurred; therefore, IACUC oversight was not required.

### Study population

The curated 2020 release of the HLES data contained 27,541 survey records collected between January 1, 2020 and December 31, 2020. In the health status section of HLES, dog owners were asked if their dogs have ever been diagnosed with various medical conditions within a set of pathophysiologic process or organ system categories. Pathophysiologic processes that often affect more than one organ system (e.g., cancer, trauma, infection, etc.) were presented at the beginning of the survey to encourage owners to record such conditions in those categories. The top five diagnostic categories reported by owners within the DAP pack in the 2020 curated dataset included dermatologic, traumatic, oral, infectious, and orthopedic disease. We identified these frequently reported disease categories as target disease categories (TDC) to be evaluated in this study.

Dogs were eligible for inclusion if they had veterinary electronic medical records (VEMRs) covering at least 85% of their life up to the time of HLES available for review. To determine an effective sample size for evaluation from among the eligible population, we conducted power analysis using Cohen’s kappa. Our analysis suggested that a sample of 300 dogs would yield 90% power to detect whether HLES reports of dogs with no diseases in the TDCs matched VEMR records with no diseases in the TDCs better than expected by chance. Thus, to adequately assess agreement between VEMR data and the owner-reported HLES for the five TDCs, a total study sample of 350 dogs was randomly selected from among those eligible – 250 dogs with a minimum of one TDC diagnosis and 100 dogs without a TDC diagnosis – with the additional 50 above the 300-dog target included to account for an anticipated 16% dropout rate due to incomplete records.

### EMR data extraction

For each dog included in this study, one of two clinical veterinarian authors (SS, AY) independently filled out a dog Health Status questionnaire identical to that in the HLES (referred to as VEMR Health Status in this report) using the dogs’ VEMR. Investigators completing the VEMR Health Status questionnaires were unaware of the owners’ original responses in the HLES Health Status questionnaire. For a condition to be coded as a diagnosis in the VEMR Health Status questionnaire, the VEMR had to contain sufficient information to support either a definitive diagnosis or a presumptive diagnosis for which treatment was initiated. For example, a diagnosis of neoplasia was made if cytology or histopathology confirmed the diagnosis, whereas a diagnosis of parvovirus was based on a positive viral PCR in the face of supportive clinical signs. Many of the diagnoses could be identified on a physical examination (e.g., heart murmur, dental calculus, medial patellar luxation) and thus were listed as a diagnosis if they were documented on at least one physical examination. If a condition was only listed as a differential in the VEMR it was not recorded as a diagnosis in the survey. As HLES includes clinical-sign based diagnoses (e.g., pruritus, lameness, alopecia) as well as more broad diagnoses in which other diagnoses might fall (e.g., granuloma, gastrointestinal parasites), diagnoses based on VEMR review were recorded to the most specific level of understanding. For example, if a dog was noted to have gastrointestinal parasites and the fecal floatation results showed roundworms, the diagnosis was recorded as “roundworms” instead of “gastrointestinal parasites” in the VEMR Health Status Questionnaire. In contrast, if a dog was noted to have pruritic skin disease but there was no evidence in the VEMR that fleas, other ectoparasites, or food allergies had been ruled out to provide a diagnosis of atopic dermatitis, the dog was classified as having “pruritus”. All HLES diagnoses identified in the VEMR, regardless of if they fell within or outside of a TDC, were recorded. To ensure that data extraction was replicable, reviewer concordance was tested on a subset of 25 randomly selected dog VEMRs. Both reviewers independently completed the VEMR Health Status questionnaires based on VEMR data for each individual in the subset, after which the completed questionnaires were compared. Upon confirming appropriate agreement [21], the remaining 325 records were reviewed and VEMR Health Status questionnaires completed. The original owner-completed HLES Health Status questionnaires (referred to as HLES Health Status in this report) and the new reviewer-completed VEMR Health Status questionnaires (referred to as VEMR Health Status in this report) were then compared.

### Statistical analyses

For each TDC we include an overall percentage agreement, numbers of agreements and disagreements, the Kappa statistic and the Gwets AC1 statistic [22]. The Kappa statistic quantifies how much the observed agreement is over-and-above what would be expected based on chance alone. If the prevalence of a condition is low, then expected agreement based on chance alone is high (0/0 agreement is high by chance), so Kappa tends to be very low. Gwets AC1 is a measure of chance-corrected agreement that is less impacted by the prevalence of the condition than Kappa. Levels of agreement statistics over 0.60 are considered to reflect substantial agreement [23]. Agreements included conditions that were reported to be present in both the HLES Health Status and VEMR Health Status as well as conditions that were reported to be not present in both the HLES Health Status and VEMR Health Status. For these analyses at the TDC level (Table 2), if any sub-condition was reported for the TDC in both HLES Health Status and the VEMR Health Status it is counted as an agreement, regardless of whether the specific condition was a match. Subsequent tables describe agreement/disagreement at the level of the specific conditions within categories.

## Results

The study population included 250 dogs for which the owners identified at least one diagnosis within five target disease categories and 100 dogs for which no diagnoses were reported in any of the target categories per HLES Health Status. Forty-one dogs were determined to have an incomplete VEMR available for review and were excluded, resulting in a total population of 309 dogs, Table 1. The study sample included 54% single-breed and 46% mixed-breed dogs. Fifty-five percent were male and 45% were female, with the vast majority of dogs in both sexes spayed or neutered. Most dogs (68%) were mature adults [24] and lived in suburban areas (65%). The majority of the owners of these dogs self-identified as White (96%), and were primarily 55 or older (63%) and educated (86% with a Bachelor’s degree or higher). Median annual household income varied, with the plurality reporting $60K-$120K (30%). Dogs and owners lived in all regions of the U.S., with the highest number (39%) in the West.

**Table 1:** Dog and owner characteristics at time of HLES for dogs included in analyses of owner-reported health conditions and conditions in VEMR. Dog Aging Project, 2020.

Human Owner Demographic Characteristics	n	%	Dog Demographic Characteristics	n	%
**Age Range**			**Breed Status**		
18–24 years	1	<1%	Single-breed	166	54%
25–34 years	26	8%	Mixed breed	143	46%
35–44 years	34	11%	**Sex/Reproductive Status**		
45–54 years	53	17%	Male	170	55%
55–64 years	102	33%	Neutered	154	91% (of males)
65 + years	93	30%	Intact	16	9% (of males)
**Education Level**			Female	139	45%
High school grad or equivalent	3	<1%	Spayed	130	94% (of females)
Some college credit	21	7%	Intact	9	7% (of females)
Associate’s degree, trade, technical or vocational training	19	6%	**Size**		
Bachelor’s degree or higher	266	86%	Small (<25 kg)	170	55%
**Annual Household Income**			Large (>25 kg)	139	45%
< $60,000	35	11%	**Life Stage**		
$60,000 - $119,999	95	31%	Puppy	3	<1%
$120,000 - $179,999	65	21%	Young adult	39	13%
>= $180,000	70	23%	Mature adult	210	68%
Prefer not to answer	44	14%	Senior	57	18%
**Race**			**Geographic** **Characteristics**		
White	296	96%	**US Region**	**n**	**%**
Black/African-American	2	<1%			
Asian	14	5%	Northeast	60	19%
American Indian	1	<1%	South	66	21%
Alaska Native/Native Hawaiian	0	0%	Midwest	60	19%
Pacific Islander	1	<1%	West	119	39%
Other	2	<1%	HI/AK	4	1%
			**Home Area Type** (self-report)		
			Urban	49	16%
			Suburban	200	65%
			Rural	60	19%

### Overall agreement between owner-reported health data (HLES) and VEMR-collected health data among target disease categories (TDCs)

**Fig 1:**
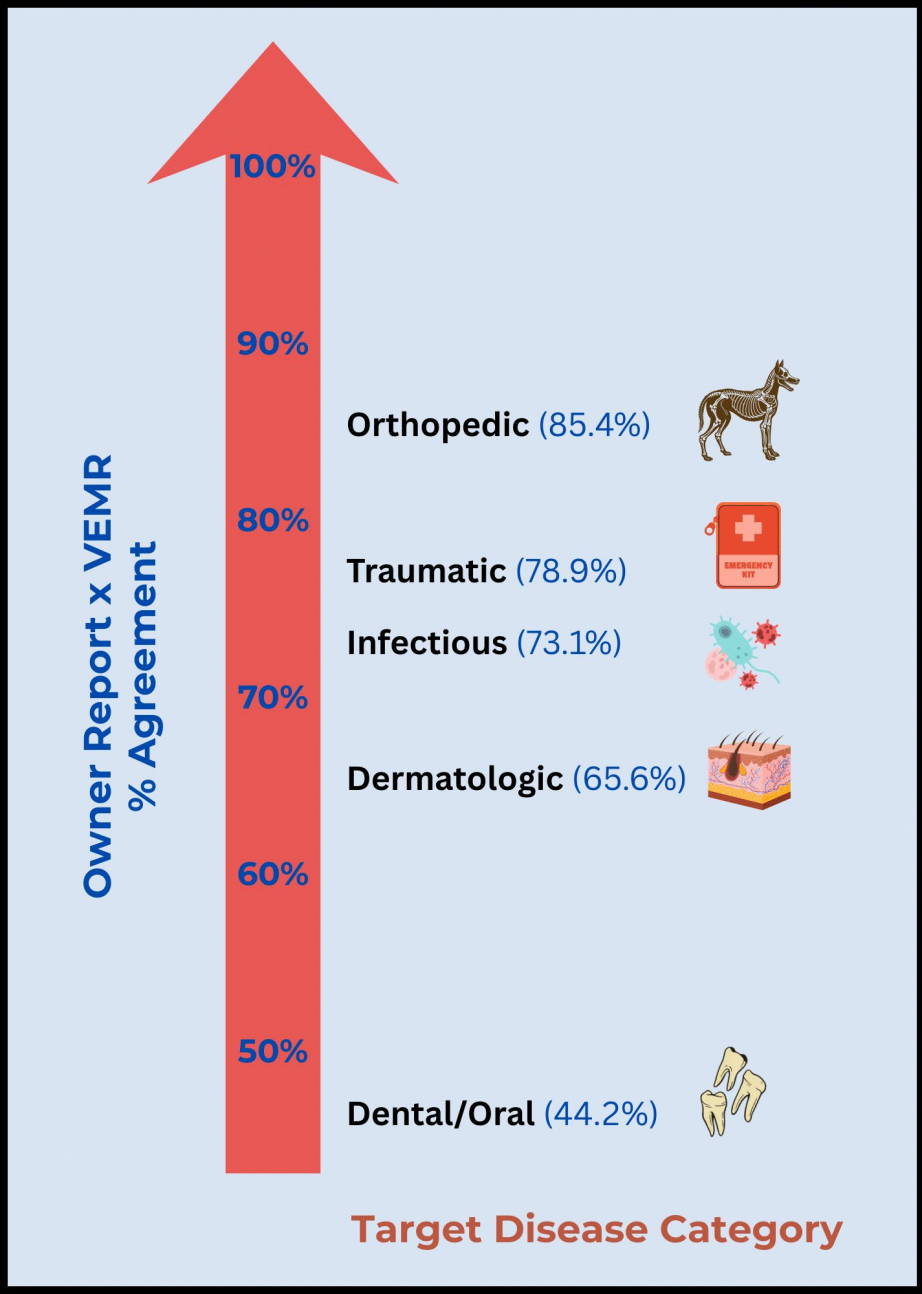
Overall agreement between owner-reported diagnoses and VEMR diagnoses for five target diseases categories.

Three of the five TDCs – Orthopedic, Traumatic, and Infectious – had overall agreement scores above 70%, and two – Dermatologic and Dental/Oral – were below 70% agreement (Fig 1). Overall agreement was highest in the Orthopedic category (85%; Gwet’s AC1 0.77), and agreement as reflected by Gwet’s AC1 indicates that only Orthopedic and Traumatic TDCs have a substantial level of agreement. Overall agreement scores were lowest for conditions classified as Dental/Oral disease (44%), followed by those classified as Dermatologic (65%), Table 2. Gwet’s AC1 likewise indicates very low agreement in these TDCs (−0.12 and 0.33, respectively). In both of these TDCs there were frequently conditions detected in VEMR Health Status that were not reported in HLES Health Status. In all but one disease category (Dental/Oral), agreement on the absence of disease was higher than agreement on the same disease being present.

**Table 2:** Accuracy of Owner-Reported Disease by Category. Positive and negative agreement results as well as Cohen’s Kappa and Gwet’s AC1 values for the comparison between owner-reported medical history in the Health, Life, and Experience Survey (HLES) and the researcher-reviewed veterinary electronic medical record (VEMR) of five target disease categories.

Disease Category	Overall Agreement n (%)	Agree disease present	Agree disease not present	HLES, not VEMR, indicates disease presence	VEMR, not HLES, indicates disease presence	Cohen’s Kappa	Gwet’s AC1
Orthopedic	263 (85.4)	53	210	11	34	0.61	0.77
Traumatic	243 (78.9)	51	192	29	36	0.47	0.65
Infectious	225 (73.1)	56	169	36	47	0.38	0.52
Dermatologic	202 (65.6)	79	123	14	92	0.34	0.33
Dental/Oral	136 (44.2)	73	63	3	169	0.13	−0.12

### Agreement within individual TDCs

**Fig 2:**
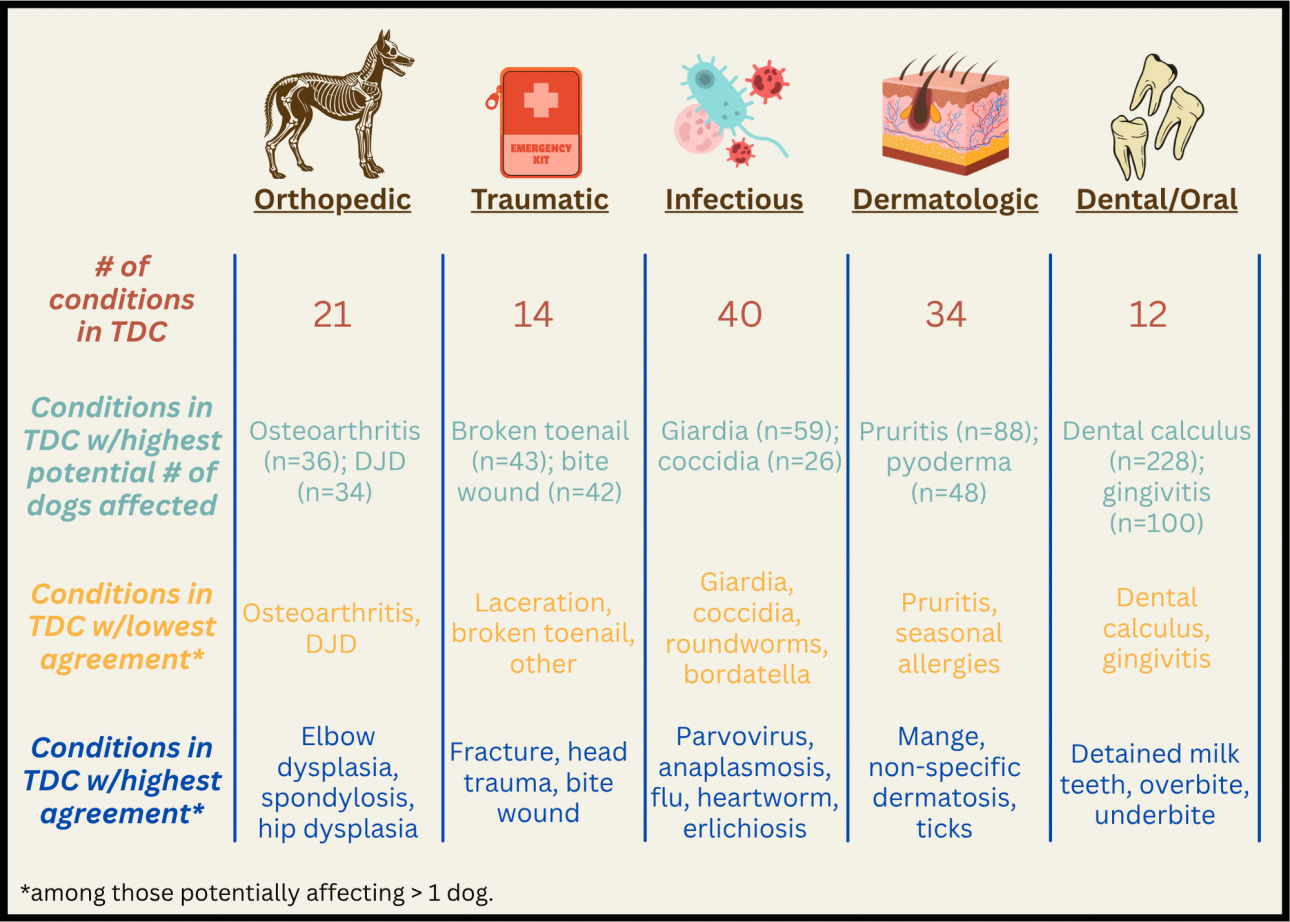
Summary of individual conditions within target disease categories.

Twenty-one individual diseases comprised the Orthopedic category, Table 3. Overall agreement was above 88% for all 21 conditions with 13 conditions (62%) having an overall agreement equal to or greater than 99%. Across the Orthopedic conditions, there was lowest agreement regarding the presence of osteoarthritis (88.3%) and degenerative joint disease (89%) (Fig 2). There was a lack of agreement regarding the presence of osteoarthritis in 36 dogs, with 30 dogs having osteoarthritis recorded only in VEMR Health Status and six dogs having osteoarthritis recorded only in HLES Health Status. Similarly, 34 dogs were recorded to have degenerative joint disease according to VEMR Health Status, with no corresponding diagnosis reported in HLES Health. Across all orthopedic disease categories, a specific orthopedic disease diagnosis was noted to be present in HLES Health Status, VEMR Health Status, or both in 187 instances. Agreement on the presence of a specific orthopedic disease was noted in 49 instances, while HLES and VEMR Health Status disagreed on the presence of a disease in 138 instances. Specifically, the VEMR Health Status indicated disease presence in 108 instances, while the HLES Health Status indicated disease presence in 30 instances.

**Table 3:** Agreement among specific conditions within Orthopedic disease category.

Orthopedic Disease	Potential # of dogs affected^ n (%)	Overall Agreement n (%)	Agree disease present	Agree disease not present	HLES, not VEMR, indicates disease presence	VEMR, not HLES, indicates disease presence
Carpal subluxation syndrome	0 (0.0)	308 (100.0)	0	308	0	0
Cruciate ligament rupture	23 (7.4)	299 (97.0)	14	285	0	9
Degenerative joint disease	36 (11.7)	274 (89.0)	2	272	0	34
Dwarfism	0 (0.0)	308 (100.0)	0	308	0	0
Elbow dysplasia	5 (1.6)	306 (99.4)	3	303	1	1
Growth deformity	0 (0.0)	308 (100.0)	0	308	0	0
Hip dysplasia	7 (2.3)	304 (98.7)	3	301	3	1
Intervertebral disc disease	8 (2.6)	301 (97.7)	1	300	2	5
Lameness (chronic/recurrent)	13 (4.2)	295 (95.8)	0	295	7	6
Osteoarthritis	49 (15.9)	272 (88.3)	13	259	6	30
Osteochondritis dissecans (OCD)	0 (0.0)	308 (100.0)	0	308	0	0
Osteomyelitis	0 (0.0)	308 (100.0)	0	308	0	0
Panosteitis	0 (0.0)	308 (100.0)	0	308	0	0
Patellar luxation	19 (6.2)	301 (97.7)	12	289	0	7
Rheumatoid arthritis	1 (0.3)	307 (99.7)	0	307	1	0
Spondylosis	3 (0.9)	305 (99.0)	0	305	1	2
Other	17 (5.5)	291 (94.5)	0	291	6	11
**Congenital**						
Missing part/whole limb	0 (0.0)	308 (100.0)	0	308	0	0
Valgus deformity	1 (0.3)	307 (99.7)	0	307	0	1
Varus deformity	1 (0.3)	307 (99.7)	0	307	0	1
Other	4 (1.3)	305 (99.0)	1	304	3	0

^Highest potential number of dogs affected by the condition based on disease presence indicated in HLES Health Status, VEMR Health Status, or both (percentage out of total n = 308 dogs).

Across all conditions classified as Traumatic, HLES and VEMR Health Status agreed that a specific trauma had occurred in 48 instances, Table 4. Disagreements were noted in 136 cases with the VEMR Health Status reporting a specific trauma in 71 instances, while the HLES Health Status indicated a specific trauma in 65 instances. The injuries with the highest potential (i.e., indicated in HLES Health Status, VEMR Health Status, or both) number of dogs affected were torn/broken toenails (n = 43) and bite wounds from other dogs (n = 42) (Fig 2). For these common injuries, agreement between HLES Health Status and VEMR Health Status for incidents of broken toenails was 90% and when disagreements were noted, HLES Health Status indicated presence (14 dogs) almost as frequently as VEMR Health Status (16 dogs). For bite wounds caused by another dog, while overall agreement was 93% (including absence), VEMR Health Status showed half the number of bite wounds (7) compared to HLES Health Status (14). The condition impacting the third highest potential number of dogs was laceration (n = 37). While overall agreement (including absence) was high (89%) for lacerations, a substantial number of owners did not report lacerations (9 reports in HLES Health Status vs. 24 reports in VEMR Health Status).

**Table 4:** Agreement among specific conditions within the Traumatic disease category.

Traumatic Disease	Potential # of dogs affected^ n (%)	Overall Agreement n (%)	Agree disease present	Agree disease not present	HLES, not VEMR, indicates disease presence	VEMR, not HLES, indicates disease presence
Bite wound from dog	42 (13.6)	287 (93.2)	21	266	14	7
Bite wound from another animal	7 (2.3)	303 (98.4)	2	301	2	3
Fall from a height	1 (0.3)	307 (99.7)	0	307	1	0
Fractured bone	7 (2.3)	305 (99.0)	4	301	2	1
Head trauma, any cause	2 (0.6)	306 (99.4)	0	306	2	0
Hit by car or other vehicle	1 (0.3)	307 (99.7)	0	307	1	0
Kicked by horse/other large animal	1 (0.3)	308 (100.0)	1	307	0	0
Laceration	37 (12.0)	275 (89.3)	4	271	9	24
Penetrating wound	7 (2.3)	302 (98.1)	1	301	3	3
Proptosis	0 (0.0)	308 (100.0)	0	308	0	0
Snakebite	1 (0.3)	308 (100.0)	1	307	0	0
Tail injury	6 (1.9)	302 (98.0)	0	302	4	2
Torn or broken toenail	43 (14.0)	278 (90.3)	13	265	14	16
Other	29 (9.4)	280 (90.9)	1	279	13	15

^Highest potential number of dogs affected by the condition based on disease presence indicated in HLES Health Status, VEMR Health Status, or both (percentage out of total n = 308 dogs).

Nearly half (19/40) of the individual diseases in the Infectious disease category were not identified in either HLES Health Status nor VEMR Health Status and were therefore reported as 100% agreement based on the absence of these diseases, Table 5. *Giardia* had the highest representation in the Infectious category, reportedly affecting up to 59 (19%) of all dogs with infectious diseases (Fig 2). Agreement between HLES Health Status and VEMR Health Status for G*iardia* was 88%; however, many owners did not report Giardia (13 dogs in HLES Health Status vs. 25 dogs in VEMR Health Status). The next most prevalent conditions were Coccidia, Bordetella, and roundworm, potentially affecting up to 26, 25, and 20 dogs, respectively. There were five reports of Coccidia in HLES Health Status, versus 19 reports in VEMR Health Status six HLES Health Status versus 13 VEMR Health Status reports indicated presence of Bordetella; and seven HLES Health Status compared to 12 VEMR Health Status reports indicated presence of roundworm.

**Table 5:** Agreement among specific conditions within the Infectious disease category.

Infectious Disease	Potential # of dogs affected^ n (%)	Overall Agreement n (%)	Agree disease present	Agree disease not present	HLES, not VEMR, indicates disease presence	VEMR, not HLES, indicates disease presence
Anaplasmosis	5 (1.6)	306 (99.4)	3	303	2	0
Aspergillosis	0 (0.0)	308 (100.0)	0	308	0	0
Babesiosis	0 (0.0)	308 (100.0)	0	308	0	0
Blastomycosis	0 (0.0)	308 (100.0)	0	308	0	0
Bordetella and/or parainfluenza	25 (8.1)	289 (93.8)	6	283	6	13
Brucellosis	0 (0.0)	308 (100.0)	0	308	0	0
Campylobacteriosis	1 (0.3)	307 (99.7)	0	307	0	1
Chagas disease (trypanosomiasis)	0 (0.0)	308 (100.0)	0	308	0	0
Coccidia	26 (8.4)	284 (92.2)	2	282	5	19
Coccidioidomycosis	2 (0.6)	307 (100.0)	1	306	1	0
Dermatophytosis (ringworm)	1 (0.3)	307 (99.7)	0	307	0	1
Distemper	0 (0.0)	308 (100.0)	0	308	0	0
Ehrlichiosis	2 (0.6)	306 (99.4)	0	306	2	0
Fever of unknown origin	2 (0.6)	306 (99.4)	0	306	1	1
Gastrointestinal parasites	8 (2.6)	300 (97.4)	0	300	4	4
Giardia	59 (19.2)	270 (87.7)	21	249	13	25
Granuloma	0 (0.0)	308 (100.0)	0	308	0	0
Heartworm infection	2 (0.6)	306 (99.4)	0	306	2	0
Histoplasmosis	0 (0.0)	308(100.0)	0	308	0	0
Hepatozoonosis	0 (0.0)	308(100.0)	0	308	0	0
Hookworms	16 (5.2)	296 (100.0)	4	292	4	8
Influenza	2 (0.6)	306 (99.4)	0	306	1	1
Isospora	3 (1.0)	305 (99.0)	0	305	0	3
Leishmaniasis	0 (0.0)	308 (100.0)	0	308	0	0
Leptospirosis	3 (1.0)	305 (99.0)	0	305	3	0
Lyme disease	9 (2.9)	301 (97.7)	2	299	7	0
MRSA/MRSP	0 (0.0)	308 (100.0)	0	308	0	0
Mycobacterium	0 (0.0)	308 (100.0)	0	308	0	0
Parvovirus	3 (1.0)	307 (99.7)	2	305	1	0
Plague (Yersinia pestis)	0 (0.0)	308 (100.0)	0	308	0	0
Pythium	0 (0.0)	308 (100.0)	0	308	0	0
Rocky Mountain Spotted Fever	0 (0.0)	308 (100.0)	0	308	0	0
Roundworms	20 (6.5)	289 (93.8)	1	288	7	12
Salmonellosis	0 (0.0)	308 (100.0)	0	308	0	0
Salmon poisoning	0 (0.0)	308 (100.0)	0	308	0	0
Tapeworms	12 (3.9)	298 (97.0)	2	296	4	6
Toxoplasma	0 (0.0)	308 (100.0)	0	308	0	0
Tularemia	0 (0.0)	308 (100.0)	0	308	0	0
Whipworms	9 (2.9)	301 (97.7)	2	299	2	5
Other	14 (4.5)	295 (95.8)	1	294	6	7

^Highest potential number of dogs affected by the condition based on disease presence indicated in HLES Health Status, VEMR Health Status, or both (percentage out of total n = 308 dogs).

HLES Health Status and VEMR Health Status agreed that disease was present in 35 instances of Dermatologic diseases, Table 6. When disagreements were noted, the VEMR Health Status indicated presence of a diagnosis in 268 instances, while the HLES Health Status indicated a diagnosis in 95 instances. Among skin-related disorders, the conditions that potentially affected the highest number of dogs were pruritus (n = 88; 29%), pyoderma or bacterial dermatitis (n = 48; 16%), seasonal allergies (n = 34; 11%), sebaceous cysts (n = 31; 10%), and chronic skin infections (n = 31; 10%). In each of these conditions there was substantial disagreement between HLES Health Status and VEMR Health Status reports regarding disease presence. Four HLES Health Status reports indicated disease presence for pruritis compared to 73 VEMR Health Status reports; two HLES Health Status compared to 46 VEMR Health Status reports indicated pyoderma or bacterial dermatitis; 27 HLES Health Status reports indicated seasonal allergies, compared to six VEMR Health Status reports; and six HLES Health Status reports indicated chronic/recurrent skin infections compared to 22 VEMR Health Status reports.

**Table 6:** Agreement among specific conditions within Dermatologic disease category.

Dermatologic Disease	Potential # of dogs affected n (%)	Overall Agreement n (%)	Agree disease present	Agree disease not present	HLES, not VEMR, indicates disease presence	VEMR, not HLES, indicates disease presence
Alopecia	14 (4.5)	296 (96.1)	2	294	2	10
Atopic dermatitis (atopy)	19 (6.2)	290 (94.1)	1	289	9	9
Chronic/recurrent hot spots	11 (3.6)	297 (96.4)	0	297	5	6
Chronic/recurrent skin infections	31 (10.1)	280 (90.9)	3	277	6	22
Contact dermatitis	1 (0.3)	307 (99.7)	0	307	1	0
Discoid lupus erythematosus	0 (0.0)	308 (100.0)	0	308	0	0
Flea allergy dermatitis	15 (4.9)	296 (96.1)	3	293	5	7
Fleas	17 (5.5)	291 (94.5)	0	291	7	10
Food/medicine allergy	15 (4.9)	298 (96.8)	5	293	6	4
Ichthyosis	1 (0.3)	308 (100.0)	1	307	0	0
Lick granuloma	7 (2.3)	301 (97.7)	0	301	0	7
Non-specific dermatosis	5 (1.6)	304 (98.7)	1	303	3	1
Panepidermal pustular pemphigus	0 (0.0)	308 (100.0)	0	308	0	0
Paraneoplastic pemphigus	0 (0.0)	308 (100.0)	0	308	0	0
Pemphigus erythematosus	0 (0.0)	308 (100.0)	0	308	0	0
Pemphigus foliaceus	0 (0.0)	308 (100.0)	0	308	0	0
Pemphigus vulgaris	0 (0.0)	308 (100.0)	0	308	0	0
Pododermatitis	25 (8.1)	283 (91.9)	0	283	0	25
Polymyositis	0 (0.0)	308(100.0)	0	308	0	0
Pruritis	88 (28.6)	231 (75.0)	11	220	4	73
Pyoderma or bacterial dermatitis	48 (15.6)	260 (100.0)	0	260	2	46
Sarcoptic mange	2 (0.6)	306 (99.4)	0	306	1	1
Seasonal allergies	34 (11.0)	275 (89.3)	1	274	27	6
Sebaceous adenitis	1 (0.3)	307 (99.7)	0	307	0	1
Sebaceous cysts	31 (10.1)	281 (91.2)	4	277	6	21
Seborrhea or seborrheic dermatitis	1 (0.3)	307 (99.7)	0	307	0	1
Systemic demodectic mange	3 (1.0)	306 (99.4)	1	305	1	1
Systemic lupus erythematosus	0 (0.0)	308 (100.0)	0	308	0	0
Ticks	7 (2.3)	302 (98.0)	1	301	4	2
Other	17 (5.5)	291 (94.5)	0	291	6	11
**Congenital**						
Dermoid cysts	0 (0.0)	308 (100.0)	0	308	0	0
Spina bifida	0 (0.0)	308 (100.0)	0	308	0	0
Umbilical hernia	5 (1.6)	304 (99.7)	1	303	0	4
Other	0 (0.0)	308 (100.0)	0	308	0	0

^Highest potential number of dogs affected by the condition based on disease presence indicated in HLES Health Status, VEMR Health Status or both (percentage out of total n = 308 dogs).

Overall agreement was the lowest (44%) in the Dental/Oral disease category compared to other TDCs, Table 2. For all diseases in this category, the VEMR and HLES Status agreed on a specific diagnosis in 84 instances. When disagreements were noted, the VEMR Health Status indicated presence of a diagnosis in 362 instances, while the HLES Health Status indicated 24 disease diagnoses, Table 7. Among all oral diseases, the conditions that potentially impacted the highest number of dogs were dental calculus (n = 228; 74%) and gingivitis (n = 100; 32%) (Fig 2). These two conditions also accounted for the highest disagreement between HLES and VEMR Health Status in the category, with dental calculus at 37% agreement and gingivitis at 69% agreement, Table 6. Notably, dental calculus was not reported in HLES Health Status when it was absent in VEMR Health Status, while VEMR Health Status reported 195 diagnoses that were not noted in HLES Health Status. Similarly, gingivitis was reported in only two HLES Health Status reports, while 93 VEMR Health Status reports indicated presence of gingivitis.

**Table 7:** Agreement among specific conditions within the Dental/Oral disease category.

Dental/Oral Disease	Potential # of dogs affected n (%)	Overall Agreement n (%)	Agree disease present	Agree disease not present	HLES, not VEMR, indicates disease presence	VEMR, not HLES, indicates disease presence
Dental calculus	228 (74.0)	113 (36.7)	33	80	0	195
Extracted teeth	50 (16.2)	280 (90.9)	22	258	11	17
Fractured teeth	53 (17.2)	270 (96.4)	15	255	6	32
Gingivitis	100 (32.5)	213 (69.2)	5	208	2	93
Masticatory myositis	0 (0.0)	308 (100.0)	0	308	0	0
Oronasal fistula	0 (0.0)	308 (100.0)	0	308	0	0
Overbite	2 (0.6)	306 (99.4)	0	306	0	2
Retained deciduous teeth	8 (2.6)	303 (98.4)	3	300	1	4
Sialocele	0 (0.0)	308 (100.0)	0	308	0	0
Underbite	11 (3.6)	300 (97.4)	3	297	0	8
Other	18 (5.8)	293 (95.1)	3	290	4	11
**Congenital**						
Cleft lip	0 (0.0)	308 (100.0)	0	308	0	0
Cleft palate	0 (0.0)	308 (100.0)	0	308	0	0
Missing teeth	0 (0.0)	308 (100.0)	0	308	0	0
Other	0 (0.0)	308 (100.0)	0	308	0	0

^Highest potential number of dogs affected by the condition based on disease presence indicated in HLES Health Status, VEMR Health Status, or both (percentage out of total n = 308 dogs).

## Discussion

In comparing owner-provided dog health data (HLES Health Status) with data extracted in a standardized manner from VEMRs (VEMR Health Status) for the same dogs, we find that dog owners who are asked to report veterinary diagnoses are generally reliable in their report of the absence of health conditions across five target disease categories, but are less reliable reporting the presence of specific diseases within categories. Existing discrepancies between owner-reported health outcomes and reviews of the VEMR data— especially among the most frequently reported disease categories (those designated here as target disease categories)— warrant consideration. To that end, we identified potential sources of such discrepancies in three areas, including the design of the survey instrument, survey end-user (owner/reporter) error, and the nature of the disease or condition. Regarding survey design, inconsistent reporting could arise in cases where the HLES instrument does not offer discrete diagnoses that are recognizable by name or description to be selected by dog owners, such as “kennel cough” not being recognized as Bordetella, or a “cut” as a laceration. Indeed, while VEMR Health Status indicated 24 instances of laceration, owners selected this diagnosis in HLES Health Status only nine times.

Lack of clarity in the HLES survey instrument on how to locate and select the correct disease category for reporting individual diagnoses could also impact owner response, leading to an inaccurate or null selection, or reporting of disease present in a category other than intended. To help owners locate and report their dogs’ medical conditions, HLES was designed with categories of medical conditions presented first, and discrete conditions provided within those categories. Some conditions that could logically fit in more than one category (e.g., discoid lupus erythematosus as both Dermatologic and Immune-Mediated) were presented in more than one category to assist the respondent in locating conditions of interest. Owners might have chosen to report the condition in either or both categories. The analysis presented here focused on categorical agreement as a first assessment and some owner-reported conditions might not have been included if they were reported in an alternate category. Future analyses of the accuracy of reporting specific disease conditions could evaluate discrete diagnoses, independent of category.

Owner self-diagnoses of dogs’ conditions and/or misinterpretation or poor recall of diagnoses determined by their veterinarian present additional room for reporting error and thus disagreement between VEMR and HLES data. For example, pruritus, a non-specific term meaning itchy skin, had the lowest agreement among listed dermatologic conditions, with the condition noted far more frequently in VEMR Health Status than HLES Health Status. In veterinary practice, the degree to which owners pursue definitive diagnosis for their dogs’ conditions varies widely. For this reason, medical conditions offered as response variables in owner-facing surveys within the DAP intentionally include both definitive diagnoses and clinical signs. A clinical sign such as pruritus could be recorded in the medical record even without an etiologic explanation but might not be recognized by an owner as a medical condition warranting reporting in a health history. A related challenge involves conditions that owners might frequently perceive as “normal”. For example, while veterinarians could be recording the presence of plaques and other dental calculus in clinical notes, owners might not recognize these conditions as true “diagnoses” or might not consider them to warrant reporting.

Conversely, when it is clear and obvious that a condition is present or absent, such as an infectious disease for which blood or stool might be tested, we see high agreement in that condition, which consequently impacts the overall category agreement. For example, Traumatic disease is a category within which most individual diagnoses have high agreement (one of two TDCs having substantial agreement per Gwet’s AC1), because it is often quite clear whether trauma occurred (e.g., getting hit by a car). Where we do see disagreement in Traumatic disease, it does not seem to be a matter of the trauma not having occurred, but rather whether it was reported. For example, bite wounds were found in HLES Health Status more often than in VEMR Health Status, which suggests owners might recognize the trauma has occurred but might not have sought care, in which case the incident would not be recorded in the VEMR. The opposite seems to be true for lacerations, wherein there were a higher number of instances in VEMR Health Status than in HLES Health Status, which implies the dog *was* seen by a veterinarian at the time of trauma, but owners did not report the laceration, perhaps because of non-recognition of the term, resolution of the problem, not considering it a medical condition, or other reasons.

High agreement among most individual conditions but low overall categorical agreement in the categories evaluated here were most frequently due in part to (1) one condition with low agreement representing the bulk of the number of dogs affected in the category (i.e., *Giardia* in the Infectious disease category), (2) fewer instances of the more definitive conditions described above, such as getting kicked by a horse (clear and specific) vs. laceration (requires interpretation) in the Trauma category, or a combination of these two.

This study was subject to potential limitations. While we have a very diverse sample of dogs distributed across the entire United States, dog owners choose to nominate their dog and therefore the data are subject to self-selection bias. Owners who tend to participate in surveys are more likely to nominate their dogs and complete the survey, possibly leading to a biased sample. Also in terms of sampling biases, the dog owner population is skewed toward being highly educated and financially stable, which may potentially impact both the degree of survey agreement and decisions around seeking veterinary care, which would ultimately affect what is reported on VEMR and thus also agreement. From a record standpoint, the owner-provided VEMR utilized in this study might not have included every veterinary visit that dog has had. To decrease gaps in medical record data, we selected a study sample of dogs that had VEMR available covering greater than 85% of their lifetime to date. In addition, most VEMRs come from primary care veterinary clinics, which might have excluded notes from emergency veterinary visits, resulting in under-reporting of traumatic and other urgent conditions in the VEMR Health Status. Furthermore, information is not always transcribed into the medical record, with one previous study revealing that only 64% of observed problems discussed during a first opinion veterinary practice consultation are recorded in the VEMR [25]. Finally, the prevalence of most diagnoses included in this study was low. Consequently, while our findings suggest that dog owners are generally reliable when reporting the absence of veterinary diagnoses, this reliability could differ in study samples with higher disease prevalence.

Compared to use of VEMR-derived dog health data, use of owner-reported dog health data presents important opportunities for large-scale longitudinal research that includes both populations that might infrequently access veterinary care, and medical conditions for which veterinary care might not be needed. The study reported here compared owner-reported and medical record dog health data using the DAP’s HLES and identified some notable areas of disagreement that might be related to term recognition, perceived importance of a medical condition, or survey design factors. Quality control is vital to getting the most out of this longitudinal dataset. Work is ongoing to modify the presentation of survey items in the Health Section instrument – such as through reviewing and editing language, clarifying descriptions of conditions, and incorporating easy reporting help functions – to improve its ability to accurately capture health information from dog owners in order to take full advantage of the data they uniquely provide.

## Conclusions

Owner-reported dog health data have the potential to be a reliable source of information for use in research. Owner-reported data showed good agreement with medical records data at the categorical level for some of the most commonly reported disease categories. Owner-reported data showed poorer agreement at the level of specific conditions within disease categories. Further adaptation of reporting mechanisms is crucial to improve the accuracy and utility of owner-reported dog health data.

## Supporting information

S1 File Results of VEMR analyses indicating presence or absence of evidence of diagnosis with specific conditions included. (XLSX)

S2 File Tabular data comparing owner-reported vs VEMR abstracted diagnoses. (XLSX)
